# Prognostic value of geriatric nutritional risk index in patients with stable coronary artery disease undergoing percutaneous coronary intervention

**DOI:** 10.1186/s12872-024-03940-w

**Published:** 2024-05-21

**Authors:** Maobin Kuang, Jiajun Qiu, Ruijuan Yang, Chao Wang, Xin Huang, Guobo Xie, Guotai Sheng, Yang Zou

**Affiliations:** 1 Jiangxi Medical College, Nanchang University, Nanchang, 330006 Jiangxi Provincial China; 2 Jiangxi Cardiovascular Research Institute, Jiangxi Provincial People’s Hospital, The First Affiliated Hospital of Nanchang Medical College, Nanchang, 330006 Jiangxi Provincial China; 3 Department of Cardiology, Jiangxi Provincial People’s Hospital, The First Affiliated Hospital of Nanchang Medical College, Nanchang, 330006 Jiangxi Provincial China; 4 Jiangxi Provincial Geriatric Hospital, Jiangxi Provincial People’s Hospital, The First Affiliated Hospital of Nanchang Medical College, Nanchang, China

**Keywords:** Stable coronary artery disease, Geriatric Nutrition Risk Index, Malnutrition, All-cause mortality, Percutaneous coronary intervention

## Abstract

**Background:**

Malnutrition increases the risk of poor prognosis in patients with cardiovascular disease, and our current research was designed to assess the predictive performance of the Geriatric Nutrition Risk Index (GNRI) for the occurrence of poor prognosis after percutaneous coronary intervention (PCI) in patients with stable coronary artery disease (SCAD) and to explore possible thresholds for nutritional intervention.

**Methods:**

This study retrospectively enrolled newly diagnosed SCAD patients treated with elective PCI from 2014 to 2017 at Shinonoi General Hospital, with all-cause death as the main follow-up endpoint. Cox regression analysis and restricted cubic spline (RCS) regression analysis were used to explore the association of GNRI with all-cause death risk and its shape. Receiver operating characteristic curve (ROC) analysis and piecewise linear regression analysis were used to evaluate the predictive performance of GNRI level at admission on all-cause death in SCAD patients after PCI and to explore possible nutritional intervention threshold points.

**Results:**

The incidence of all-cause death was 40.47/1000 person-years after a mean follow-up of 2.18 years for 204 subjects. Kaplan-Meier curves revealed that subjects at risk of malnutrition had a higher all-cause death risk. In multivariate Cox regression analysis, each unit increase in GNRI reduced the all-cause death risk by 14% (HR 0.86, 95% CI 0.77, 0.95), and subjects in the GNRI > 98 group had a significantly lower risk of death compared to those in the GNRI < 98 group (HR 0.04, 95% CI 0.00, 0.89). ROC analysis showed that the baseline GNRI had a very high predictive performance for all-cause death (AUC = 0.8844), and the predictive threshold was 98.62; additionally, in the RCS regression analysis and piecewise linear regression analysis we found that the threshold point for the GNRI-related all-cause death risk was 98.28 and the risk will be significantly reduced when the subjects' baseline GNRI was greater than 98.28.

**Conclusions:**

GNRI level at admission was an independent predictor of all-cause death in SCAD patients after PCI, and GNRI equal to 98.28 may be a useful threshold for nutritional intervention in SCAD patients treated with PCI.

**Supplementary Information:**

The online version contains supplementary material available at 10.1186/s12872-024-03940-w.

## Background

SCAD is a widespread cardiovascular disease, featured pathologically as the presence of atherosclerotic plaques within the walls of the coronary arteries [1, 2]. Atherosclerotic plaques can cause severe stenosis or even rupture of coronary vessels, leading to cardiovascular events like angina pectoris, ischemic heart failure, and acute myocardial infarction, which can lead to death in severe cases [3, 4]. Globally, CAD is a major cause of death, especially among the elderly population [5, 6]. PCI is, currently, one of the main invasive treatments for patients with CAD, which can effectively relieve myocardial ischemia and improve myocardial oxygenation, and reduce angina attacks and myocardial infarction [7, 8]. Previous studies have shown that the presence of malnutrition may increase the incidence of post-PCI complications and cardiovascular events in elderly SCAD patients, which has a significant impact on the long-term prognosis of patients [9, 10]; in addition, malnutrition can induce a chronic inflammatory response in the body, and long-term chronic inflammation can further aggravate malnutrition and oxidative stress, thereby exacerbating endothelial dysfunction and forming a malnutrition-inflammation-atherosclerosis syndrome [11, 12]. Therefore, evaluating the nutritional conditions of elderly SCAD patients undergoing PCI during hospitalization may have important prognostic implications.

GNRI is a nutrition-related index that can be applied to evaluate the nutritional conditions of hospitalized elderly patients and to predict morbidity and mortality from malnutrition-related diseases in elderly patients developed by Bouillanne O et al. [13]. Currently, several studies have reported the association of nutritional conditions assessed using GNRI with the occurrence and prognosis of CAD and the risk of CAD comorbidities [10, 11, 14–17]. Also, the association of GNRI with the long-term prognosis of CAD patients was explored in a study by Wada H et al., who concluded that lower GNRI was an independent risk factor for all-cause death in post-PCI CAD patients [18]. However, the predictive performance of GNRI at the time of admission for elective PCI in newly diagnosed SCAD patients for the future occurrence of all-cause death and the possible threshold points for nutritional interventions are unclear. Therefore, this study aimed to investigate the risk assessment/predictive value of GNRI levels at admission in elderly patients with newly diagnosed SCAD for the occurrence of all-cause death after PCI and to further search for possible thresholds of nutritional intervention.

## Methods

### Research design and ethics approval

This study conducted a secondary analysis using inpatient examination data and post-discharge follow-up data of newly diagnosed SCAD patients treated with elective PCI at Shinonoi General Hospital from October 2014 to October 2017. All information regarding patient enrollment has been described in detail elsewhere [19]. In brief, the research team from Shinonoi General Hospital enrolled hospitalized patients who were newly diagnosed with SCAD and underwent selective PCI treatment at the hospital between 2014 and 2017. After excluding patients with a history of old myocardial infarction and those with malignant tumors, a total of 204 subjects were finally included; and the researcher collected data on various tests during the hospitalization of these patients and continued to follow the patients after their discharge. In a previous study, Suzuki S et al. used the data of this cohort of patients to investigate the association of serum albumin (ALB) with the occurrence of major adverse cardiac events, and uploaded the complete data of all subjects to the Daryd database [20]; additionally, Suzuki S et al. declared that all enrolled patients and their written informed consent were approved by Shinonoi General Hospital Ethics Committee, and the subjects’ identifying information was anonymized [19].

On this basis, the current study used data from this cohort to explore the risk assessment and predictive value of GNRI measured at admission for the onset of all-cause death in patients with SCAD undergoing PCI; since this study was a secondary analysis of data from previous research and all data on subjects had been anonymized, Jiangxi Provincial People’s Hospital Ethics Committee had authorized this study and dispensed with repeatedly obtaining written informed consent from subjects (IRB: 2022-006). All procedures in the current study were following the Declaration of Helsinki [21].

### Data collection and definitions

During the subjects’ hospitalization, the researchers collected extensive data on all subjects including clinical characteristics [sex, body mass index (BMI), weight, age, height, diastolic blood pressure (DBP), estimated glomerular filtration rate (eGFR), systolic blood pressure (SBP), estimated glomerular filtration rate (eGFR), smoking history, Multivessel PCI, history of previous stroke, history of diabetes], type of coronary lesions (calcified lesions, ostial lesion, bifurcation lesions, chronic coronary obstruction lesions), laboratory measurements [total cholesterol (TC), low-density lipoprotein (LDL-C), ALB, Glycohemoglobin (HbA1c), triglycerides (TG), high-density lipoprotein (HDL-C), C-reactive protein (CRP), hemoglobin (Hb)], echocardiography (left ventricular ejection fraction) and angiography data. Where ALB was measured using the bromocresol purple assay and the LABOSPECT 008 analyzer and anthropometric indicators were measured using standard methods. SCAD was diagnosed based on angiographic findings of ≥ 90% epicardial coronary stenosis or findings of ≥ 75% epicardial coronary stenosis with symptoms of exercise-induced chest pain or clinical evidence of myocardial ischemia induced by stress testing; PCI procedures and coronary angiography were performed in all subjects according to guidelines and standard protocols, and subjects were administered thienopyridines and aspirin prior to the procedure [19]. The primary follow-up endpoint event in our study was the occurrence of all-cause death, and the event was validated by reviewing the medical record data of treated patients.

### Calculation of the GNRI

The marker used to evaluate nutritional conditions in this study was GNRI, which was calculated as GNRI = 1.489 × ALB (g/L) + 41.7 × actual weight (kg)/ideal weight (kg). Where ALB and actual weight were measured while the subjects were hospitalized, and ideal weight was calculated using the Lorenz formula, i.e., height (cm)-100- [height (cm)-150]/4 for men and height (cm)-100-([height (cm)-150]/2.5) for women. When the calculation result of the ideal weight < actual weight, the actual weight/ideal weight was considered as one; additionally, we defined GNRI ≤ 98 as the presence of malnutrition risk and GNRI > 98 as the absence of malnutrition risk according to the findings of the GNRI developer Bouillanne O et al. [13].

### Statistical analysis

All statistical analyses for this study were completed on R Language 3.4.3 and Empower(R) 4.1 software and significance was set at two-tailed *P* < 0.05.

Information about subjects was described by grouping them according to their GNRI values at baseline and whether death occurred during follow-up, respectively. Measurement data with normal and nonnormal distributions were expressed by mean (standard deviation) and median (lower quartile, upper quartile), respectively, while count data were described using frequency (%). In the inter-group comparison of measurement data, two independent sample t-test was used for variables with normal distribution and the Kruskal-Wallis H test for nonnormal distribution; while the chi-square test was used for inter-group comparisons of count data.

The Kaplan-Meier curves were used to evaluate the cumulative incidence risk of all-cause death of subjects in different GNRI groups, and the log-rank test was used to compare between groups; in addition, we also determined whether the proportional risk hypothesis was valid by observing whether the Kaplan-Meier curves intersected between different groups [22]. Before building the Cox regression model, we also checked whether there was collinearity between GNRI and other covariates using multiple linear regression analysis, and covariates with a final variance inflation factor greater than 5 were defined as collinear variables and excluded from model adjustment [23]. In the Cox regression analysis, we included GNRI as a continuous variable and a categorical variable in the model and calculated the hazard ratio (HR) and 95% confidence interval (CI) after stepwise adjustment for other non-collinear variables [24]. First, an unadjusted Crude Model was established to initially assess the association of GNRI with all-cause death risk; then in Model 1, sex, age, height, and BMI were adjusted; Model 2 was adjusted for blood lipid and blood glucose parameters (TG, HbA1c, LDL-C, HDL-C) on the basis of Model 1; finally, Model 3 further adjusted for all remaining non-collinear variables based on Model 2. Additionally, to further explore the shape of the association, we also fitted a dose-response relationship curve based on the multivariable-adjusted Model 3 using a RCS regression model with 4 knots and then used the piecewise linear regression model to automatically calculate the possible best inflection point on the curve by a recursive method.

Based on Model 3 we also explored the possible effect of the types of subjects’ coronary lesions [calcified lesions, ostial lesions, bifurcation lesions, and chronic total obstruction (CTO) lesions] on the association of GNRI with all-cause death risk and checked whether the effect of various lesion types on the association was significant using the log-likelihood ratio test. Finally, we also plotted the ROC curve to explore the predictive performance of GNRI and BMI for all-cause death and recorded the area under the ROC curve (AUC) and the prediction threshold, and the predictive accuracy of both was compared using the Delong test.

## Results

### Clinical baseline characteristics

The average age of the 204 subjects in the current study was 72.6 years, with 62 (30.39%) women and 142 (69.61%) men. During 443.89 person-years (mean 2.18 years) of follow-up, eighteen deaths occurred with an incidence density of 40.55/1000 person-years. The subjects’ basic characteristics were shown in Tables 1 and 2. Table 1 shows comparative information on the baseline characteristics of subjects at risk of malnutrition (GNRI ≤ 98) compared to those at no risk of malnutrition (GNRI > 98). The results showed that subjects without risk of malnutrition were younger and had a higher prevalence of smokers and also had higher levels of height, weight, BMI, ALB, eGFR, TC, TG, LDL-C, SBP, DBP, Hb, LVEF and lower CRP levels than those with risk of malnutrition, but there were no significant differences in sex composition, proportion of complex PCI procedures, history of stroke, proportion of diabetic patients, HDL-C and HbA1c levels and types of coronary lesion. In Table 2, we further compared the clinical characteristics of subjects who died during the follow-up period with those who did not. Our analysis revealed that those in the dead group tended to exhibit characteristics of malnutrition, as they were generally older and had significantly lower height, LDL-C, weight, TG, GNRI, BMI, eGFR, TC, Hb, and ALB levels and had higher CRP levels and proportion of smokers, while there were also no significant differences in sex composition, proportion of complex PCI procedures, history of stroke, proportion of diabetic patients, HDL-C, HbA1c, SBP, DBP, LVEF levels, and types of coronary lesion. These results suggested clinical indicators that may be associated with GNRI and that the occurrence of death may be related to the risk of malnutrition.

**Table 1:** Baseline characteristics of subjects grouped by GNRI

	GNRI ≤ 98	GNRI > 98	*P*-value
Number of subjects, n (%)	78 (38.23%)	126 (61.77%)	
Sex, n (%)			0.302
Female	27 (34.62%)	35 (27.78%)	
Male	51 (65.38%)	91 (72.22%)	
Age, years	78.00 (72.00–85.00)	70.00 (65.00-76.75)	< 0.001
Height, cm	157.00 (150.00-163.75)	160.50 (154.00-167.00)	< 0.001
Weight, kg	52.70 (45.00-57.40)	65.00 (57.12-71.00)	< 0.001
BMI, kg/m^2^	20.92 (18.69–23.47)	24.58 (22.73–26.43)	< 0.001
ALB, g/L	35.00 (31.25-37.00)	42.00 (41.00–44.00)	< 0.001
eGFR, mL/min/1.73m^2^	56.50 (33.25–68.75)	67.00 (59.00-76.75)	< 0.001
TC, mg/dL	171.50 (148.75-197.25)	191.00 (171.50–212.00)	< 0.001
TG, mg/dL	91.50 (59.00-132.00)	129.00 (90.50–183.00)	< 0.001
HDL-C, mg/dL	49.00 (39.25-57.00)	49.00 (42.00–57.00)	0.840
LDL-C, mg/dL	94.00 (81.00-115.00)	116.00 (98.00-135.00)	< 0.001
HbA1c, %	5.90 (5.60–6.50)	6.00 (5.70–6.73)	0.443
SBP, mmHg	132.00 (119.25-145.75)	140.00 (126.50–147.00)	0.014
DBP, mmHg	73.50 (66.00-84.75)	78.00 (71.00–86.00)	0.042
LVEF, %	65.30 (60.25-68.00)	70.00 (65.00-76.75)	0.026
Hb	12.30 (11.10-13.38)	14.50 (13.62–15.40)	< 0.001
CRP	0.22 (0.07–0.85)	0.09 (0.04–0.18)	< 0.001
Calcified lesions, n (%)			0.203
No	70 (89.74%)	105 (83.33%)	
Yes	8 (10.26%)	21 (16.67%)	
Ostial lesions, n (%)			0.848
No	67 (85.90%)	107 (84.92%)	
Yes	11 (14.10%)	19 (15.08%)	
Bifurcation lesions, n (%)			0.564
No	37 (47.44%)	65 (51.59%)	
Yes	41 (52.56%)	61 (48.41%)	
CTO, n (%)			0.113
No	76 (97.44%)	116 (92.06%)	
Yes	2 (2.56%)	10 (7.94%)	
Multivessel PCI			0.369
No	55 (70.51%)	96 (76.19%)	
Yes	23 (29.49%)	30 (23.81%)	
OCI			0.551
No	62 (79.49%)	107 (84.92%)	
Yes	16 (20.51%)	19 (15.08%)	
Smoking history			0.006
No	49 (62.82%)	54 (42.86%)	
Yes	29 (37.18%)	72 (57.14%)	
DM			0.353
No	47 (60.26%)	84 (66.67%)	
Yes	31 (39.74%)	42 (33.33%)	
All cause death			< 0.001
No	61 (78.21%)	125 (99.21%)	
Yes	17 (21.79%)	1 (0.79%)	

Values were expressed as mean (standard deviation) or medians (quartile interval) or n (%)

*Abbreviations:* *GNRI* Geriatric nutritional risk index, *BMI* Body mass index, *ALB* Albumin, *eGFR* Estimated glomerular filtration rate, *TC* Total cholesterol, *TG* Triglycerides, *HDL-C* High-density lipoprotein, *LDL-C* Low-density lipoprotein, *HbA1c* Glycohemoglobin, *SBP* Systolic blood pressure, *DBP* Diastolic blood pressure, *LVEF* Left ventricular ejection fraction, *Hb* Hemoglobin, *CRP* C-reactive protein, *CTO* Coronary total occlusion, *OCI* Old Cerebral Infarction, *DM* Diabetes mellitus

**Table 2:** Baseline characteristics of subjects grouped by death endpoint

	Non-death	Death	*P*-value
Number of subjects, n (%)	186 (91.17%)	18 (8.83%)	
Sex, n (%)			0.776
Female	56 (30.11%)	6 (33.33%)	
Male	130 (69.89%)	12 (66.67%)	
Age, years	72.00 (65.25-79.00)	81.00 (77.00-87.75)	< 0.001
Height, cm	160.00 (152.25–166.00)	154.00 (149.25–161.50)	0.029
Weight, kg	60.75 (53.00-69.75)	48.50 (41.50–53.00)	< 0.001
GNRI	101.26 (95.07-105.73)	84.26 (77.26–93.57)	< 0.001
BMI, kg/m^2^	23.79 (21.35–25.79)	19.98 (18.19–21.89)	< 0.001
ALB, g/L	41.00 (37.25-43.00)	32.50 (27.50-35.75)	< 0.001
eGFR, mL/min/1.73m^2^	65.00 (54.25-76.00)	55.00 (33.50-64.75)	0.012
TC, mg/dL	187.00 (168.00-208.00)	163.00 (144.50–180.00)	0.008
TG, mg/dL	119.00 (84.00-160.00)	88.00 (61.25–141.00)	0.174
HDL-C, mg/dL	49.00 (41.50–57.50)	46.50 (37.25-50.00)	0.132
LDL-C, mg/dL	110.00 (91.75–130.00)	89.50 (75.75–108.00)	0.033
HbA1c, %	6.00 (5.70–6.77)	5.80 (5.40–6.30)	0.702
SBP, mmHg	137.50 (123.25–147.00)	134.00 (120.25–146.50)	0.941
DBP, mmHg	76.00 (70.00–85.00)	76.50 (66.25–90.50)	0.848
LVEF, %	66.00 (62.20–68.00)	64.70 (60.27-68.00)	0.110
HB	14.00 (12.60–15.10)	11.25 (10.17–12.90)	< 0.001
CRP	0.11 (0.04–0.25)	0.49 (0.19–1.65)	0.042
Calcified lesions, n (%)			0.693
No	159 (85.48%)	16 (88.89%)	
Yes	27 (14.52%)	2 (11.11%)	
Ostial lesions, n (%)			0.652
No	158 (84.95%)	16 (88.89%)	
Yes	28 (15.05%)	2 (11.11%)	
Bifurcation lesions, n (%)			0.622
No	94 (50.54%)	8 (44.44%)	
Yes	92 (49.46%)	10 (55.56%)	
CTO, n (%)			0.267
No	174 (93.55%)	18 (100.00%)	
Yes	12 (6.45%)	0 (0.00%)	
Multivessel PCI			0.855
No	138 (74.19%)	13 (72.22%)	
Yes	48 (25.81%)	5 (27.78%)	
OCI			0.551
No	155 (83.33%)	14 (77.78%)	
Yes	31 (16.67%)	4 (22.22%)	
Smoking history			0.015
No	89 (47.85%)	14 (77.78%)	
Yes	97 (52.15%)	4 (22.22%)	
DM			0.209
No	117 (62.90%)	14 (77.78%)	
Yes	69 (37.10%)	4 (22.22%)	

Values were expressed as mean (standard deviation) or medians (quartile interval) or *n* (%). Other abbreviations as in Table 1

**Fig. 1:**
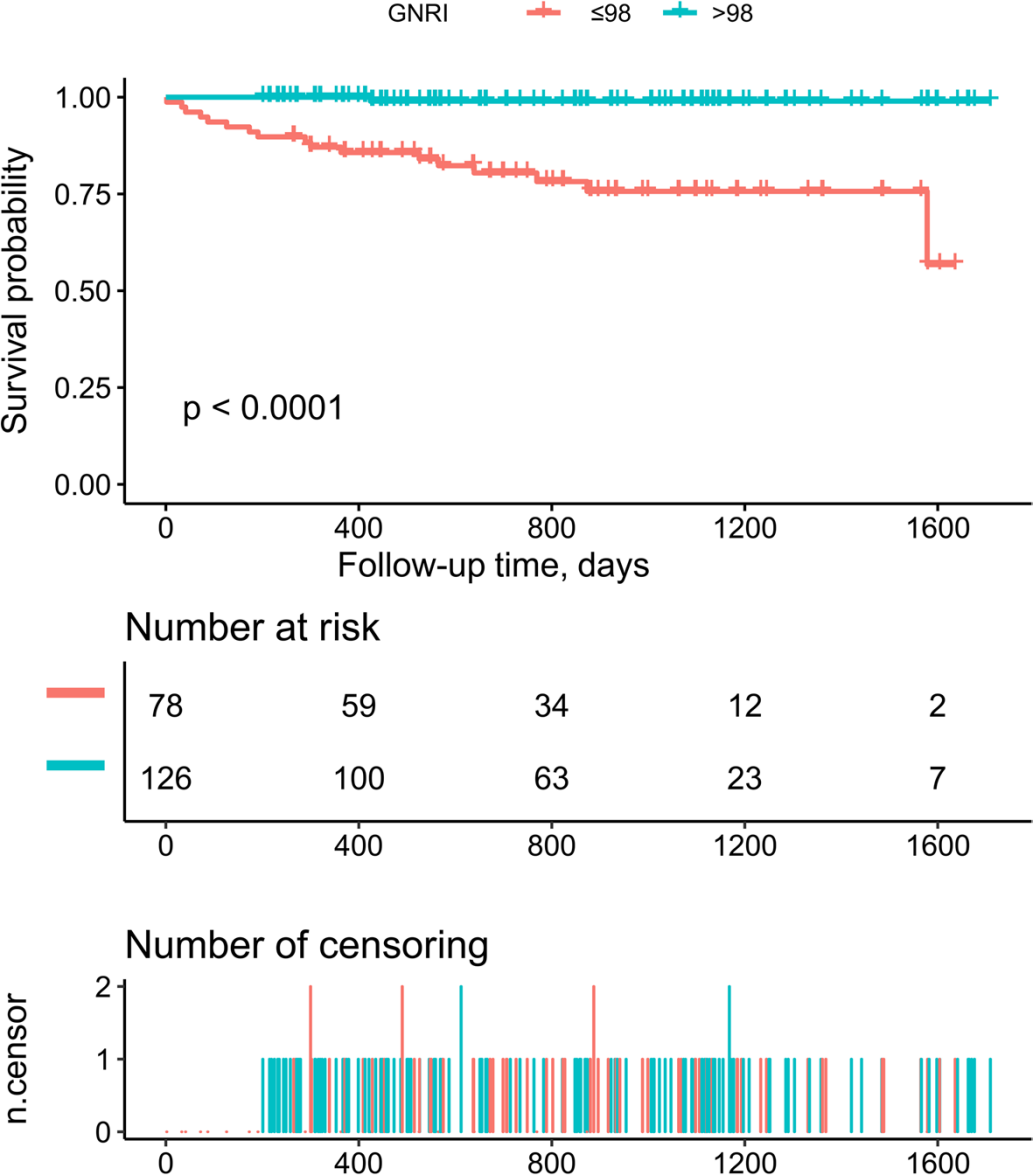
Kaplan-Meier analysis for all-cause death risk according to groups of nutritional status

Figure 1 shows the Kaplan-Meier survival curves of the GNRI ≤ 98 group and the GNRI > 98 group, and it can be seen that subjects in the group of GNRI ≤ 98 had significantly higher mortality than the GNRI > 98 group (log-rank *P* < 0.0001), and the curves between the two groups did not intersect indicating that the proportional hazard assumption was met in the current study.

### Association between GNRI and all-cause death risk

Before establishing the multivariate-adjusted Cox regression models, the collinearity screening analysis screened out the collinear variables weight, ALB, and TC with GNRI, and they will not be brought into the model adjustment. Table 3 shows the results of the association analysis between GNRI and all-cause death risk showing that in the unadjusted Crude Model, GNRI was negatively associated with all-cause death risk, either as a continuous variable (HR 0.86, 95% CI 0.82, 0.91; *P* < 0.0001) or as a categorical variable (HR 0.03, 95% CI 0.00, 0.25; *P* = 0.001); and the negative correlation between GNRI and all-cause death risk was slightly attenuated after stepwise adjustment for multiple confounders in Models 1, 2, and 3. According to the results of Model 3, each 1 unit increase in GNRI was associated with an 14% reduced risk of all-cause death (HR 0.86, 95% CI 0.77, 0.95; *P* = 0.0041); in addition, patients without risk of malnutrition had a significantly lower all-cause death risk compared to those with risk of malnutrition (HR 0.04, 95% CI 0.00, 0.89; *P* = 0.0417).

**Table 3:** Association of baseline GRNI with risk of all-cause death

	HR (95% CI)							
	Crude model	*P*-value	Model 1	*P*-value	Model 2	*P*-value	Model 3	*P*-value
GNRI (continue)	0.86 (0.82, 0.91)	< 0.0001	0.88 (0.83, 0.95)	0.0006	0.87 (0.82, 0.93)	< 0.0001	0.86 (0.77, 0.95)	0.0041
GNRI (category)								
GRNI ≤ 98	Reference		Reference		Reference		Reference	
GRNI > 98	0.03 (0.00, 0.25)	0.0010	0.07 (0.01, 0.66)	0.0206	0.07 (0.01, 0.61)	0.0156	0.04 (0.00, 0.89)	0.0417

*Abbreviations: HR* hazard ratios; *CI* confidence interval; other abbreviations as in Table 1

Crude model was not adjusted

Model 1 was adjusted for sex, age, BMI, and height

Model 2 was adjusted for sex, age, BMI, height, TG, HDL-C, LDL-C, and HbA1c

Model 3 was adjusted for sex, age, BMI, height, TG, HDL-C, LDL-C, HbA1c, SBP, DBP, LEVF, and eGFR, calcified lesions, ostial lesions, bifurcation lesions, CTO, OCI, multivessel PCI, smoking history, HB, DM, CRP

### Nonlinear association analysis between GNRI and all-cause death risk

**Fig. 2:**
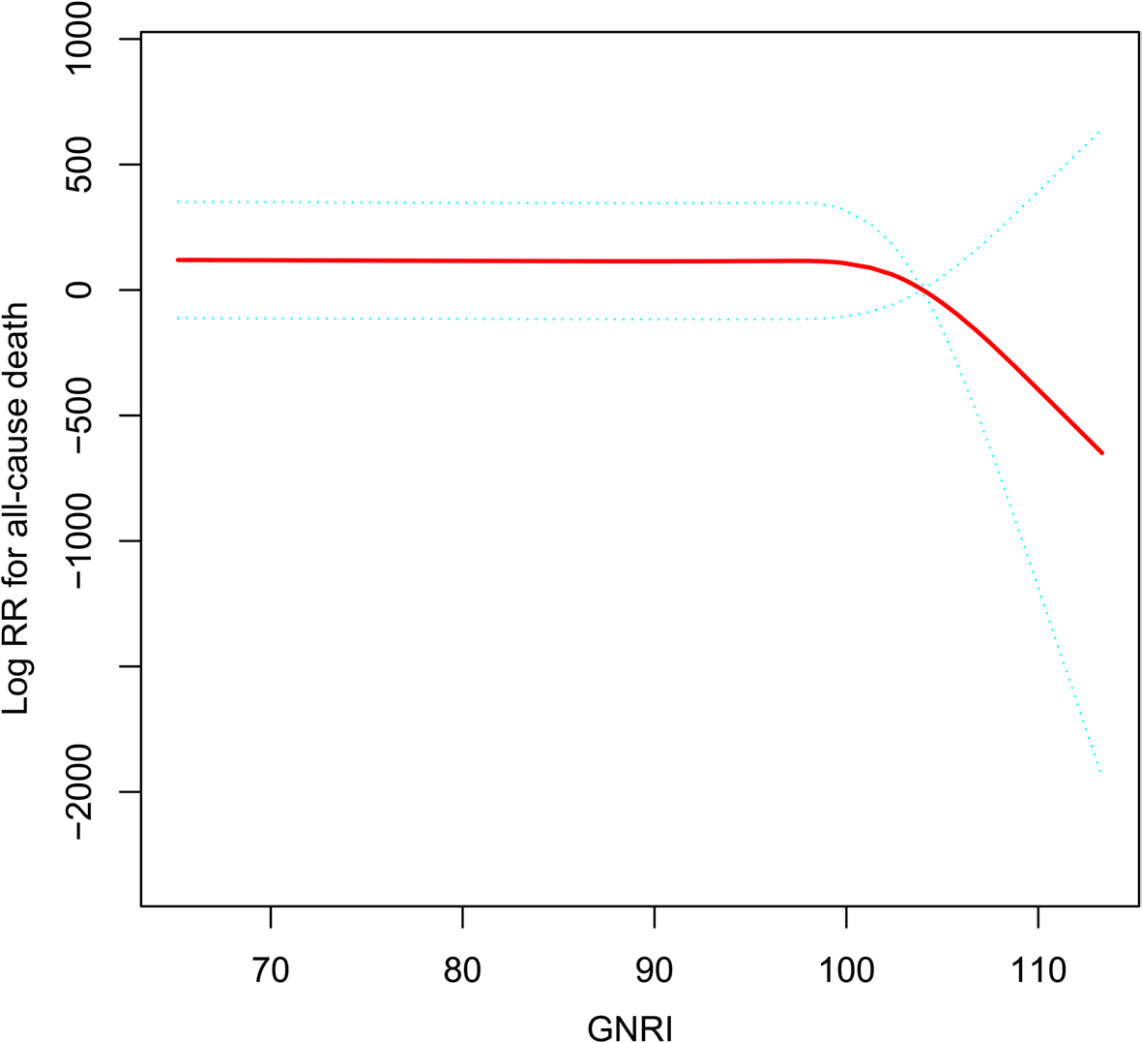
Restricted cubic spline regression analysis of the association between GNRI and all-cause death risk. *GNRI* Geriatric Nutrition Risk Index

Figure 2 shows the dose-response relationship curve of GNRI with all-cause death risk. We found that when GNRI was at a low level, the dose-response relationship curve was relatively flat and an increase in GNRI did not seem to significantly reduce all-cause death risk, whereas when GNRI reached to 95–100 interval, there was a clear inflection point on the curve and a significant reduction in GNRI-related all-cause death risk occurred after the inflection point. On this basis, we further calculated the best inflection point on the curve by piecewise linear regression analysis, and the results showed that the correlation of GNRI with all-cause death risk had the most significant change on both sides of the GNRI value equal to 98.28 (Log-likelihood ratio test *P* = 0.023) (Table 4). The HR for the association between GNRI and all-cause death risk was 0.87 (95% CI 0.76, 0.99; *P* = 0.0402) when the GNRI was less than 98.28, whereas the association was not significant at GNRI greater than 98.28, which may be attributable to the fact that there were no deaths among the subjects with a GNRI greater than 98.28 at admission in the current study. Therefore, the risk threshold on this dose-response relationship curve for all-cause death risk associated with GNRI may be located at 98.28, and GNRI equal to 98.28 may be the target value for nutritional interventions to reduce all-cause death risk in post-PCI SCAD patients.

**Table 4:** Threshold analysis of GNRI-related risk of all-cause death

	Risk of all-cause death	
	HR (95%CI)	*P*-value
Fitting model by multivariable Cox regression		
	0.86 (0.77, 0.95)	0.0041
Fitting model by two-piecewise linear regression		
The best inflection point of GNRI	98.28	
< inflection point	0.87 (0.76, 0.99)	0.0402
> inflection point	0.00 (0.00, Inf)	0.9715
Log-likelihood ratio test		0.023

*Abbreviations: HR* hazard ratios, *CI* confidence interval, *Inf* infinity

### Subgroup analysis

Considering that the types of coronary lesions in subjects may have an impact on the GNRI-related all-cause death risk, we also conducted stratified analyses based on whether the presence of calcified lesions, ostial lesions, bifurcation lesions, and CTO lesions in the subjects, and the results showed that these types of coronary lesions had no significant impact on the association between GNRI and all-cause death risk (All *P* for interaction > 0.05) (Table 5).

**Table 5:** Association of GRNI with risk of all-cause death in subgroups of coronary lesion types

Complication	HR (95%CI)	*P*-value	*P* for interaction
Ostial lesion			0.0675
No	0.84 (0.74, 0.94)	0.0027	
Yes	1.10 (0.86, 1.40)	0.4455	
Calcification			0.3087
No	0.87 (0.78, 0.97)	0.0133	
Yes	0.74 (0.51, 1.06)	0.0958	
Bifurcation lesion			0.8900
No	0.86 (0.76, 0.97)	0.0172	
Yes	0.85 (0.74, 0.98)	0.0250	
CTO			1.0000
No	0.86 (0.77, 0.95)	0.0041	
Yes	1.11 (0.0, Inf)	0.9999	

*Abbreviations: HR* hazard ratios, *CI* confidence interval, other abbreviations as in Table 1

### The predictive value of GNRI and BMI for future all-cause death event

**Fig. 3:**
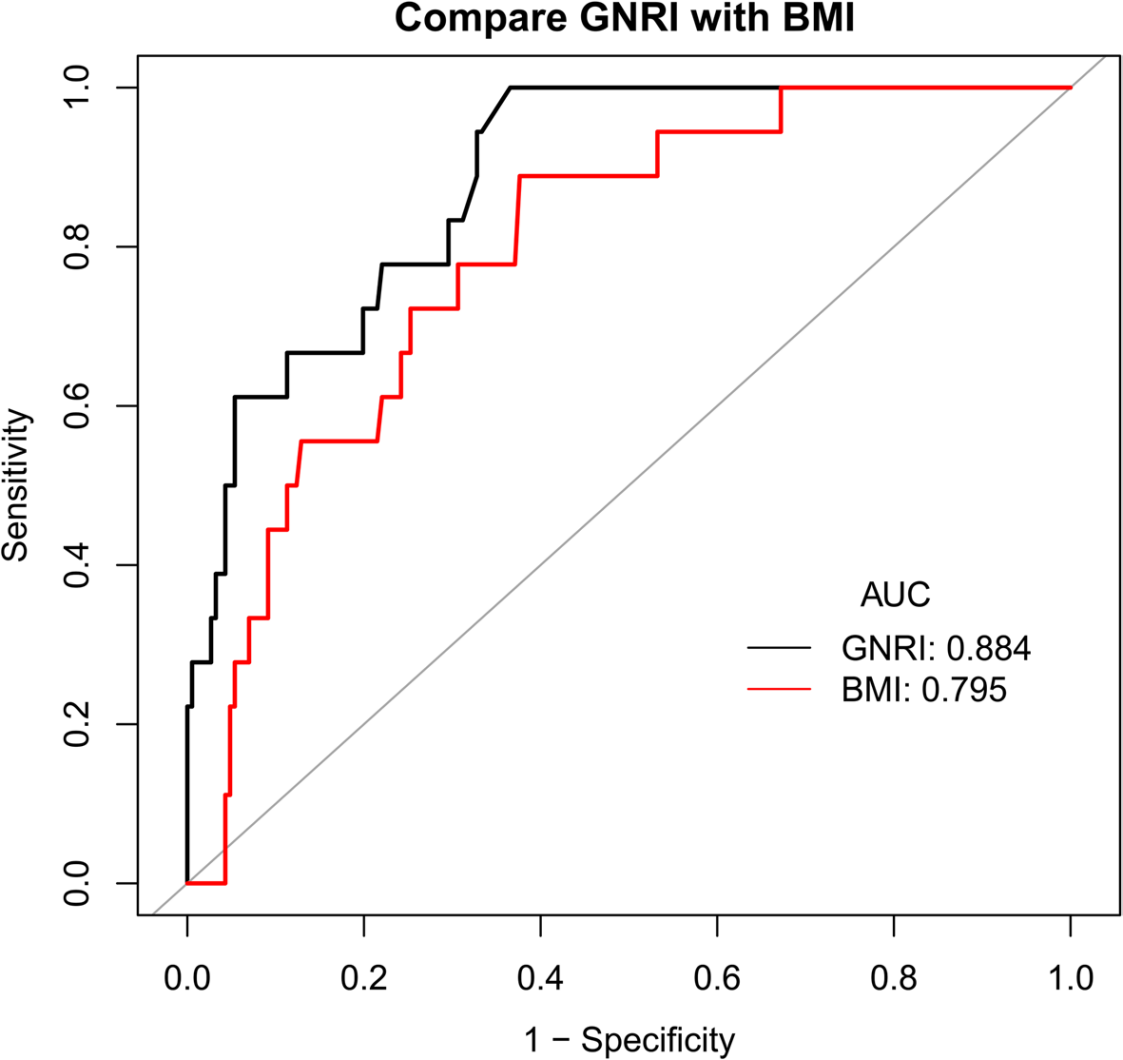
Receiver operating characteristic curve analyses for predicting all-cause death events using GNRI and BMI. *GNRI* Geriatric Nutrition Risk Index; *BMI* body mass index

In Fig. 3, we plotted ROC curves for assessing the predictive performance of baseline GNRI and BMI for future all-cause death events and found that GNRI measured on admission had excellent predictive accuracy for all-cause death events occurring in SCAD patients after PCI. When the prediction threshold of GNRI was 98.62, the AUC reached the highest 0.8844 (0.8184, 0.9505), the sensitivity and specificity at this time were 100% and 63.44%, respectively, and the Youden index was 0.6344; the extremely high sensitivity of this prediction threshold point may imply that all-cause death risk after PCI was significantly reduced when patients with new-onset SCAD have a GNRI above 98.62 during hospitalization. The AUC value for BMI was 0.7945, and after comparing with the Delong test, it was found that GNRI’s predictive accuracy for all-cause mortality after PCI in SCAD patients was significantly higher than that of BMI (*P* = 0.0232). Moreover, it is worth noting that the results of the ROC analysis were consistent with the results of the nonlinear analysis described above and that the optimal risk threshold point we selected in the dose-response curve was at GNRI equal to 98.28, which was also extremely close to the prediction threshold point of 98.62.

## Discussion

The results of this cohort study suggested that the GNRI of elderly patients with newly diagnosed SCAD measured on admission was associated with all-cause death risk after PCI and that a high level of GNRI was a protective factor against the occurrence of all-cause death. In addition, GNRI also had very high predictive performance (AUC = 0.8844) for the occurrence of all-cause death events in SCAD patients after PCI, with an appropriate prediction threshold of 98.62. It is worth mentioning that in the RCS regression analysis and threshold effects analysis, we found that a GNRI value equal to 98.28 was the threshold point for the GNRI-related risk of all-cause death, a threshold point that was extremely close to the prediction threshold of 98.62 in the ROC analysis and was also extremely close to the threshold point recommended by Bouillanne O et al. for no risk of malnutrition (GNRI = 98). Therefore, to reduce all-cause death risk, controlling GNRI above 98.28 may be the nutritional intervention threshold for SCAD patients admitted for PCI.

CAD, one of the most common cardiovascular diseases worldwide, is usually caused by insufficient coronary artery blood supply [1, 25, 26]. With the global aging population, unhealthy lifestyles, and the global prevalence of metabolic diseases like obesity, diabetes, and hypertension, CAD has become a major cause of death in middle-aged and elderly populations in both developed and developing countries [5, 27–29]. Recently, there has been great clinical progress in both the pharmacological treatment of CAD and invasive PCI treatment, especially since the successive introduction of drug-eluting stents and bioresorbable stents, the efficacy and safety of PCI treatment have been significantly improved [11, 30, 31]. Nevertheless, the clinical benefit of PCI therapy in SCAD patients seems to be limited to the relief of angina, reduction of the incidence of spontaneous myocardial infarction, and improvement of the survival quality of patients, without a significant impact on the long-term prognosis of SCAD patients, i.e., mortality [7, 32, 33], and therefore a large number of investigators are working to find clinical indicators that play an important role in the long-term prognosis of SCAD patients. Previous studies have shown that malnutrition is a common problem among hospitalized patients, and it is estimated that approximately 30-60% of hospitalized patients are at risk for malnutrition, with a higher prevalence in older hospitalized patients [14, 34]. The presence of malnutrition risk may further weaken the immune system, and increase the cardiac burden and risk of infection in elderly patients with cardiovascular disease thereby prolonging hospitalization, with negative and far-reaching effects on clinical outcomes in elderly patients with SCAD [35–37].

The GNRI is a simple and practical nutrition-related risk index, which evaluates nutritional conditions through three routinely measured parameters of height, weight, and ALB in hospitalized patients, and can be used for risk assessment and risk stratification of morbidity and mortality from diseases related to nutritional status in elderly patients [13]. In a previously published meta-analysis by Fan Y et al., they evaluated for the first time a total of 9277 CAD patients in eight cohort studies and analyzed the predictive value of GNRI for all-cause death or major adverse cardiac events in CAD patients, showing that GNRI was an independent predictor of all-cause death and major adverse cardiac events in patients with stable and acute CAD [38]. Moreover, the prognostic value of GNRI has been demonstrated in several studies; for example, Zhao Q et al. showed that lower GNRI was an important predictor of poor prognosis in patients with non-ST-segment elevation myocardial infarction treated with PCI [16], while Anzaki K et al. and Cheng L et al. considered that low GNRI was not only an independent risk factor for all-cause mortality and coronary artery calcification in CAD patients after PCI but also an independent predictor of long-term adverse cardiac events in CAD patients who have developed CTO lesions after PCI [10, 11]. Similar to Anzaki K et al. [11], our results showed that GNRI levels at admission in new-onset SCAD patients were significantly associated with all-cause death risk after PCI, even after adjustment of many clinical indicators like age, sex, BMI, and cardiac function parameters, and that better nutritional status was associated with a significantly lower risk of all-cause death (HR 0.86, 95% CI 0.77, 0.95; *P* = 0.0041); furthermore, by looking at Tables 1 and 2, the clinical characteristics of subjects at risk of malnutrition (GNRI ≤ 98) were very similar to the characteristics of subjects who had died during follow-up, both having higher age and CRP levels and having lower height, weight, GNRI, BMI, ALB, eGFR, TC, TG, LDL-C levels, further highlighting the relationship between nutritional status and clinical outcomes in SCAD patients after PCI. However, in contrast to the findings of Cheng L et al. [10], in the stratified analyses of this study, we found that the type of coronary artery lesions did not appear to have a significant effect on the association of GNRI with all-cause death risk (all *P* for interaction > 0.05), and no significant association was found between GNRI and all-cause death risk in the subgroup of subjects who developed CTO lesions, although this may be due to the small number of subjects (*n* = 12) who developed CTO lesions.

An innovative finding of this study was the discovery of a threshold point of nutritional intervention that may need to be reached when hospitalizing patients with newly diagnosed SCAD for elective PCI. On the one hand, in the ROC analysis of the current study, we found that GNRI had a very high predictive value for all-cause death events occurring in SCAD patients after PCI treatment and found the maximum AUC value of 0.8844 at a prediction threshold of 98.62 with 100% sensitivity and 63.44% specificity, and the very high sensitivity implying a very low false-negative rate [39]; this may mean that newly diagnosed SCAD patients with a GNRI above 98.62 on admission have a significantly lower risk of all-cause death after PCI. On the other hand, the results of the RCS regression analysis and piecewise linear regression analysis in this study further corroborated the reliability of this prediction threshold. We observed a significant inflection point in the interval 95 < GNRI < 100 on the dose-response curve of GNRI with all-cause death risk (Fig. 2), and further calculated the GNRI value of 98.28 for the inflection point by piecewise linear regression analysis (Log-likelihood ratio test *P* = 0.023). When GNRI was less than 98.28, increasing GNRI can significantly reduce all-cause death risk (HR 0.87, 95% CI 0.76, 0.99; *P* = 0.0402), whereas when GNRI was greater than 98.28, the association of GNRI with all-cause death risk was not statistically significant, but this result may be due to the fact that none of the subjects in the current study population with a GNRI greater than 98.28 on admission had died during the follow-up period. It is also worth noting that the risk threshold of 98.28 was also extremely close to the threshold value of GNRI = 98 recommended by Bouillanne O, the developer of GNRI, who defined GNRI > 98 as the threshold value for no malnutrition risk based on the threshold values of ALB and weight loss in the elderly [13]. In a word, after a comprehensive evaluation of the risk assessment and predictive performance of GNRI for the occurrence of all-cause death events in SCAD patients after PCI, we suggested that GNRI = 98.28 may be the most appropriate threshold point for nutritional intervention in elderly patients with newly diagnosed SCAD who are hospitalized for elective PCI. Clinicians should also assess the nutritional status of elderly SCAD patients by calculating GNRI while treating them with PCI, and inpatients with GNRI less than 98.28 should be given appropriate nutritional support to minimize the risk of poor prognosis after PCI [40].

The strength of this study lies in the use of rigorous statistical approaches that, after stepwise adjustment for a large number of clinical indicators, identified GNRI as an independent predictor of the occurrence of all-cause death events in newly diagnosed SCAD patients after PCI, and proposed for the first time a threshold point of nutritional intervention that may need to be achieved when hospitalizing elderly patients with newly diagnosed SCAD for elective PCI treatment. Certainly, there are several limitations: (1) The study population was relatively small and from a single center, and the follow-up period was relatively short; (2) The GNRI levels of the subjects in this study were assessed only once and were not repeatedly measured during the follow-up period; it would provide more useful information if it could be further evaluated whether an increase in GNRI during the follow-up period has an improving effect on the prognosis of SCAD patients; (3) The current study is a retrospective cohort study and there may be some residual confounding because some clinical indicators with important effects on all-cause death risk could not be collected [41]; furthermore, this study was unable to update information regarding the make and manufacturer of the machines used for coronary angiography and PCI. (4) The mean age of the subjects in the current study cohort was 72.59 years, so the results of the current study may be more appropriate for the elderly population, and further research is needed on the applicability to other age groups. (5) This study only compared the predictive performance of the basic nutritional assessment indicator, BMI, for all-cause mortality risk with GNRI. It lacks comparison data between GNRI and other nutritional assessment scores or models. Further refinement is needed in future studies. (6) All subjects in this study underwent PCI treatment. Although the PCI procedures for SCAD patients were relatively consistent, differences in surgical techniques or operator proficiency could still lead to varying treatment efficacy, potentially resulting in residual confounding.

## Conclusion

The current study found that the nutritional status of SCAD patients, as assessed by GNRI at admission, was an independent predictor of all-cause death events after PCI. We also identified a GNRI threshold of 98.28, which may be an appropriate point for nutritional intervention in newly diagnosed elderly SCAD patients who are hospitalized for elective PCI. Clinicians can improve the long-term prognosis of SCAD patients by moderately increasing their protein and calorie intake if they fall below this nutritional threshold.

### Supplementary Information

Supplementary Material 1.

## Data Availability

The data used in this study have been uploaded to the "Dryad" database (https://doi.org/10.5061/dryad.fn6730j).

## Abbreviations

GNRI: Geriatric Nutrition Risk Index
PCI: Percutaneous coronary intervention
SCAD: Stable coronary artery disease
RCS: Restricted cubic spline
ROC: Receiver operating characteristic curve
ALB: Albumin
BMI: Body mass index
DBP: Diastolic blood pressure
eGFR: Estimated glomerular filtration rate
SBP: Systolic blood pressure
TC: Total cholesterol
LDL-C: Low-density lipoprotein
BbA1c: Glycohemoglobin
TG: Triglycerides
HDL-C: High-density lipoprotein
HR: Hazard ratio
CI: 95% confidence interval
CTO: Chronic total obstruction
