# Supplementary material for: Prognostic value of geriatric nutritional risk index in patients with stable coronary artery disease undergoing percutaneous coronary intervention
Source: BMC Cardiovasc Disord. 2024 May 21;24:264. doi: 10.1186/s12872-024-03940-w (PMC11106886; doi:10.1186/s12872-024-03940-w)
Supplement: Supplementary file 1 — Supplementary Material 1. [file 12872_2024_3940_MOESM1_ESM.docx]

Supplementary Table 1: Collinearity diagnostics steps.

|  | Variance inflation factor | | | |
| --- | --- | --- | --- | --- |
|  | Step 1 | Step 2 | Step 3 | Step 4 |
| GNRI | 22.9 | 22.9 | 2.6 | 2.4 |
| Age | 2.5 | 2.5 | 2.5 | 2.5 |
| Sex | 3.1 | 3.1 | 3.1 | 3 |
| Height | 44.9 | 3.7 | 3.7 | 3.6 |
| Weight | 105.2 | NA | NA | NA |
| BMI | 65.2 | 2.6 | 1.7 | 1.6 |
| eGFR | 1.5 | 1.5 | 1.5 | 1.4 |
| Hb | 2.4 | 2.4 | 2.3 | 2.2 |
| Smoking history | 1.5 | 1.5 | 1.5 | 1.5 |
| Ostial lesions | 1.3 | 1.3 | 1.3 | 1.2 |
| Multivessel PCI | 1.6 | 1.6 | 1.6 | 1.5 |
| Bifurcation lesions | 1.4 | 1.4 | 1.3 | 1.2 |
| Calcified lesions | 1.2 | 1.2 | 1.2 | 1.2 |
| CTO | 1.3 | 1.3 | 1.3 | 1.2 |
| ALB | 20.3 | 20.3 | NA | NA |
| DM | 2.3 | 2.3 | 2.3 | 1.4 |
| OCI | 1.2 | 1.2 | 1.2 | 1.2 |
| TC | 20.1 | 19.9 | 19.8 | NA |
| TG | 4.6 | 4.6 | 4.6 | 1.4 |
| HDL-C | 5.8 | 5.8 | 5.6 | 1.4 |
| LDL-C | 13.1 | 12.9 | 12.8 | 1.4 |
| HbA1c | 2.1 | 2.1 | 2.1 | 1.1 |
| SBP | 2.6 | 2.6 | 2.6 | 2.4 |
| DBP | 2.8 | 2.8 | 2.8 | 2.6 |
| LVEF | 1.2 | 1.2 | 1.2 | 1.2 |

Note-1: Variance inflation factor = 1/(1-R^2^). Abbreviations as in Table 1.

Note-2: The variables with Variance inflation factor >5 will be regarded as collinear variables and cannot be included in the multiple regression model.
